# Blood tests – too much of a good thing

**DOI:** 10.1080/02813432.2022.2104436

**Published:** 2022-08-18

**Authors:** Henrik L. Jørgensen, Bent S. Lind

**Affiliations:** a Department of Clinical Biochemistry, Copenhagen University Hospital, Hvidovre, Denmark; b Department of Clinical Medicine, University of Copenhagen, Copenhagen N, Denmark

For many years, health care expenditure has only gone one way – up! According to an analysis by Statistics Denmark, public health care spending has increased by 46% from 2000 to 2017 compared to a rise of 15% in overall public spending in Denmark. In 2016, the US spent 17.8% of its gross domestic product on health care [1]. There are, of course, many factors driving this continuous increase in health costs: Ageing populations, expensive new treatments, increased administrative costs, etc.

Although laboratory expenditure often represents less than 5% of most hospital budgets, the impact is far-reaching given that laboratory tests influence nearly 60–70% of all medical decisions [2]. The true cost of blood testing is therefore considerably higher if you include the subsequent downstream testing and procedures, unnecessary hospitalizations, prolongation of hospitalizations, prescription of medical treatments, etc. caused by randomly abnormal blood tests.

Commonly, normal ranges are determined by examining a sufficiently large sample of healthy individuals, setting the ranges to include 95% of this sample within the normal ranges. This means, that if you analyze one biochemical parameter in a healthy individual, the risk of it being outside the normal range is 5%, purely by chance. If you analyze several, independent biochemical parameters in the same healthy individual, the risk of at least one of these parameters being abnormal is 1–0.95*^N^*, where *N* is the number of tests. The risk of spurious abnormal results reaches close to 50% when *N* is 13. In practice, the individual biochemical parameters are not completely independent, and *N* must therefore be slightly higher than 13 to reach the 50% chance of at least one abnormal result.

The frequency of testing of the 10 most frequently requested blood tests in the Capital Region of Denmark during a 10-year period from 2010 to 2019 increased overall by 8% per year corresponding to a doubling time of 9 years. The increase was more pronounced in hospital departments (11.5% per year, doubling time 6.5 years) than for GPs (3.9% per year, doubling time 18 years) [3].

There are several reasons for this large increase including ageing population, expensive new treatments as well as factors such as defensive medicine [4]. However, the major driver of the increase in the number of requested blood tests is the design of the order entry systems, most importantly the increasing size of test ordering profiles, that the physician can invoke with only a few clicks in the electronic requisition system. In many laboratories, most of the total production of test results is requested through such profiles. In the Capital Region of Denmark, the GPs can, for example, order a profile for dementia containing 25 individual biochemical tests with a risk of up to 72% of at least one abnormal result. The largest profile, used by GPs for patients with unspecific symptoms suspected of serious disease, contains 40 individual biochemical tests with a risk of up to 87% of at least one abnormal result. In many instances, these profiles will yield several abnormal results purely by chance which may lead to unnecessary further testing, treatment or hospitalization.

To give an example of the magnitude of this problem, in the Capital Region of Denmark with a population of 1.8 million people (2019), more than 2.5 million requests (from hospitals and GPs combined) for P-sodium are filed per year. More than a third of the population has a measurement of P-sodium at least once per year.

So, what can be done to stem this tsunami of blood tests?

First of all, we hope that education and an increased awareness of the overuse of blood tests may help. As physicians, we need to ask ourselves every time we order a diagnostic test (the problem applies to all paraclinical examinations) if we really need this particular test and what consequences the result of the test will have for the diagnostic process or the treatment of our patient. We do not feel confident that these questions are asked every time unnecessary tests are requested. Education may reduce the number of tests, but the effect may be more pronounced by addition of other strategies.

In laboratories, several such strategies are possible [5]. These include the design of the order entry system. Soft stops are pop-up messages in the electronic requesting systems informing the requesting physicians, e.g. that the same test was performed on the patient a short while ago, that the test only applies to a restricted type of patient or asking the physician to supply the reason for ordering the test. We used this latter approach to try to reduce the number of vitamin D analyses requested by GPs. Since the early 2000s, this analysis has seen an exponential growth making it an obvious target for this type of intervention. From January 2017, we introduced a compulsory pop-up form in which the general practitioners had to state the indication for measuring vitamin D, choosing from a predefined set of indications. This resulted in a drop of 25% of the requisitions of vitamin D [6]. Whether this decrease is permanent remains to be seen.

Other possible strategies for the laboratory are removal of obsolete tests from the order entry system, hard stops which reject the requisition automatically if certain parameters are met and implementation of minimal retesting intervals. In a study of hemoglobin A1C, another test which has seen a massive increase in use, we calculated that cancelling all follow-up measurements ordered within 16 weeks could reduce the number of hemoglobin A1C measurements by 23% [7].

Finally, economic incentive structures might also be a helpful instrument for the laboratories.

Overuse of blood tests carry not only significant direct and indirect costs for society but can also be harmful to patients eliciting unnecessary extra, potentially harmful procedures. Clinicians and laboratories must therefore work closely together to reduce overuse and to further evidence-based use of laboratory medicine.
